# Quantitative Trait Locus Mapping Combined with RNA Sequencing Reveals the Molecular Basis of Seed Germination in Oilseed Rape

**DOI:** 10.3390/biom11121780

**Published:** 2021-11-27

**Authors:** Kunjiang Yu, Yuqi He, Yuanhong Li, Zhenhua Li, Jiefu Zhang, Xiaodong Wang, Entang Tian

**Affiliations:** 1 Department of Agronomy, College of Agriculture, Guizhou University, Guiyang 550025, China; kjyu678@163.com (K.Y.); yqhe1972@163.com (Y.H.); liyuanh31@163.com (Y.L.); lixing_19841014@126.com (Z.L.); 2 Key Laboratory of Cotton and Rapeseed, Ministry of Agriculture and Rural Affairs, Institute of Industrial Crops, Jiangsu Academy of Agricultural Sciences, Nanjing 210014, China; jiefu_z@163.com

**Keywords:** *Brassica napus*, seed germination, quantitative trait locus, transcriptome, molecular basis

## Abstract

Rapid and uniform seed germination improves mechanized oilseed rape production in modern agricultural cultivation practices. However, the molecular basis of seed germination is still unclear in *Brassica napus*. A population of recombined inbred lines of *B. napus* from a cross between the lower germination rate variety ‘APL01’ and the higher germination rate variety ‘Holly’ was used to study the genetics of seed germination using quantitative trait locus (QTL) mapping. A total of five QTLs for germination energy (GE) and six QTLs for germination percentage (GP) were detected across three seed lots, respectively. In addition, six epistatic interactions between the QTLs for GE and nine epistatic interactions between the QTLs for GP were detected. *qGE.C3* for GE and *qGP.C3* for GP were co-mapped to the 28.5–30.5 cM interval on C3, which was considered to be a novel major QTL regulating seed germination. Transcriptome analysis revealed that the differences in sugar, protein, lipid, amino acid, and DNA metabolism and the TCA cycle, electron transfer, and signal transduction potentially determined the higher germination rate of ‘Holly’ seeds. These results contribute to our knowledge about the molecular basis of seed germination in rapeseed.

## 1. Introduction

In seed plants, seed germination is essential for the production of offspring and the maintenance of the species. Rapid and uniform seed germination enhances mechanized crop production in modern agricultural cultivation practices. Seed germination begins with water uptake and ends up with the emergence of the radicle from the surrounding seed tissues [1]. From a genetic point of view, seed germination is controlled by complex interactions, including those between plant hormone signal transductions, reactive oxygen species (ROS) signaling pathway, and methionine metabolism.

A pair of key hormone molecules operate during seed germination: abscisic acid (ABA) and gibberellins (Gas). ABA promotes dormancy and Gas promote germination. The two have antagonistic effects and inhibit each other’s metabolism and signaling genes [2]. In addition to ABA and Gas, other hormones or signaling molecules, such as ethylene (ETH), brassinolide (BR), cytokinin (CK), auxin (IAA), jasmonic acid (JA), salicylic acid (SA), and oxidized lipids also play roles in seed germination [3,4,5,6,7,8,9,10]. Similar to ABA and Gas, the effects of these hormones are often interactive. This interactive molecular regulatory network leads to synergistic and antagonistic effects between these hormones during seed germination. For example, Gas, ETH, and BR promote seed germination while ABA inhibits it, so that Gas, ETH, and BR are all antagonistic to the effect of ABA [3].

As signal molecules, ROS participate in the regulation of seed dormancy release, endosperm relaxation, and storage mobilization [11,12]. Excessive accumulation of ROS creates toxic molecules, which inhibit germination, in which case the antioxidant system will be activated to eliminate ROS [11]. During seed germination and seedling development, ROS may inhibit the transport of ABA from the cotyledons to embryos and in turn promote germination [13]. ROS may also stimulate the signaling and/or synthesis of Gas and change the ABA/Gas threshold to promote seed germination [14]. In addition, the interactions between ROS and other hormones, including CK, SA, JA, and ETH, have also been reported [15,16].

Obstruction of methionine (Met) synthesis can delay seed germination in *Arabidopsis* and inhibit seedling growth, which can be partially restored by adding Met to the delayed germination seeds, demonstrating that Met is involved in regulating seed germination [17]. Methionine synthase 1 activates GLR3.5 Ca^2+^ channels and regulates seed germination by promoting Met synthesis in *Arabidopsis* [18]. As an active Met, S-adenosylmethionine (AdoMet) is involved in multiple methylation reactions regulating seed germination, including the modification of DNA methylation [19] and chromosome remodeling [20]. Plant SABATH methyltransferase regulates seed germination by methylating the N-position or O-position of the hormone to reduce its activity level in the seed [21,22,23]. AdoMet is the precursor of a variety of substances related to seed germination, such as ETH, Biotin, and Polyamine [24,25]. Methionine sulfoxide is reduced to Met by methionine sulfoxide reductase to determine the lifespan of seeds [26]. In addition, cysteine (Cys) and aspartic acid (Asp) are precursors of Met and are widely involved in the regulation of seed germination [27]. Cys is also the precursor of the antioxidant glutathione (GSH) that participates in multiple metabolic processes during seed germination, including the GSH and ascorbic acid cycles [28], forming S-nitroglutathione, a storage substance for NO [29].

*Brassica napus* (AACC, 2n = 38) is an oil crop that is widely cultivated all over the world. Rapid and uniform seed germination is important for mechanized precision seeding in oilseed rape production practices. Cultivating oilseed rape varieties with good seed germination characteristics is an important means to promote rapid and uniform crop establishment. In recent years, studies have begun to focus on seed germination and have identified many quantitative trait loci (QTLs) that regulate seed germination in *B. napus*. Twenty-six QTLs related to seed germination have been found on chromosome A01–A06 and A09–A10, explaining 7.5–27.2% of the phenotypic variation (PV) [30]. Co-localization of phenotypic QTLs with expression QTLs indicated that *BrFLC2* and *BrFAD2* might be potential candidate genes regulating seed germination [30]. Genome-wide association mapping identified 20 loci related to germination performance in oilseed rape seeds, and three promising candidate genes [31]: *B. napus* orthologs of the *Arabidopsis thaliana* genes *SNOWY COTYLEDON 1*; *ARABIDOPSIS TWO-COMPONENT RESPONSE REGULATOR*; and *ARGINYL-t-RNA PROTEIN TRANSFERASE 1*. Proteomic dissection identified 17 genes corresponding to 11 differentially expressed proteins in *B. napus* [32]. These genes are within or near the associated linkage disequilibrium regions related to previously reported quantitative seed germination and vigor traits, and may regulate seed germination through protein and amino acid metabolism, and glycolysis/gluconeogenesis and energy generation [32]. QTL *GR.9.1* on chromosome A09, and *GR.11.2* on chromosome C01, explained 2.5% and 6.3% of the PV in seed germination rate, respectively [33]. An integrative approach generating transcriptomic, metabolic, and hormonal data at different stages of seed imbibition discovered that the levels of malate and aspartate metabolites were closely related to germination performance and that hormonal balance between ABA, GAs and IAA at crucial time points during seed germination might underlie seed germination differences in the accessions under study [34]. Although some genetic loci and candidate genes have been reported, the molecular basis of seed germination is still unclear in *B. napus*, and very few candidate genes have been identified.

In this study, a population (denoted the AH-RILs population) of recombined inbred lines (RILs) of *B. napus* from a cross between the lower germination rate variety ‘APL01’ and the higher germination rate variety ‘Holly’ was used to study the genetics of seed germination using QTL mapping. Pre-germinated seeds (denoted A0 and H0, respectively) and germinated seeds (denoted A1 and H1, respectively) of ‘APL01’ and ‘Holly’ were collected for RNA sequencing. The sequence variation of genes within the QTL confidence interval (CI) between ‘APL01’ and ‘Holly’ was identified using whole-genome re-sequencing technology. The aims were: (1) to detect QTLs that regulate seed germination in the whole genome; (2) to reveal the transcriptomic basis of the difference in seed germination between ‘APL01’ and ‘Holly’; and (3) to identify promising candidate genes regulating seed germination. This study will help to establish a better understanding of the molecular basis of seed germination in *B. napus*.

## 2. Results

### 2.1. Phenotypic Variation in Germination Energy (GE) and Germination Percentage (GP)

**Figure 1:**
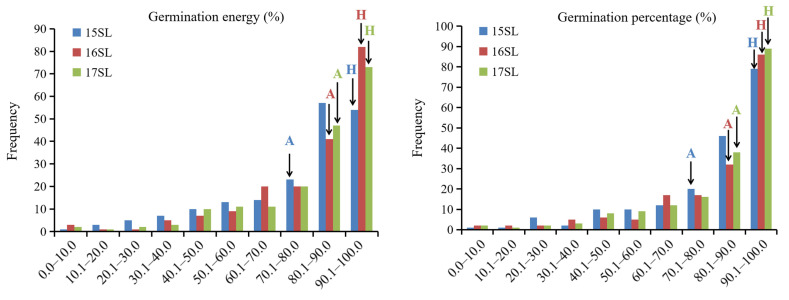
The frequency distribution of germination energy and germination percentage in the AH population. ‘A‘ represents ‘APL01’. ‘H‘ refers to ‘Holly’. 15SL, 16SL, and 17SL refer to the seed lot harvested in 2015, 2016, and 2017, respectively.

Comparative analyses showed that the GE and GP of ‘APL01’ in three SLs were significantly lower than those of ‘Holly’ (Table 1, Figure 1). In the AH-RILs population, both GE and GP exhibited a continuous negatively skewed distribution (Figure 1), with skewness ranging from −1.76 to −1.26 (Table 1). The negative super-parental phenomenon in these two traits was observed in all three SLs (Table 1, Figure 1). The mean *h^2^* of GE and GP in the three SLs were 80.2% and 80.0%, respectively (Table 1). In summary, these results indicate that both GE and GP are quantitative traits controlled by multiple genes, and that the major gene has obvious effects. QTL mapping can effectively reveal the genetic control of these two traits.

### 2.2. QTL Mapping for GE and GP

Regarding GE, a total of seven identified QTLs were detected in the three SLs with explained variances ranging from 2.04–13.61% (Table 2). They were distributed on chromosomes A4, C3 and C7. Comparative analyses found that the three identified QTLs, *iqGE/15SL.C3* (28.5–30.5 cM), *iqGE/16SL.C3* (28.5–30.5 cM) and *iqGE/17SL.C3* (28.5–29.5 cM), were co-mapped to the 28.5–30.5 cM interval on C3. These three identified QTLs were integrated into one consensus QTL (*qGE.C3*) using QTL meta-analysis. *qGE.C3* explained 8.28%, 10.59%, and 13.61% of the PV in the three SLs (15SL—the seed lot harvested in 2015, 16SL—the seed lot harvested in 2016, and 17SL—the seed lot harvested in 2017), respectively, and was considered to be the major QTL. The remaining four specific QTLs, identified in only one SL, were also treated as four consensus QTLs in the statistical analysis, namely *qGE.A4*, *qGE.C2-1*, *qGE.C2-2*, and *qGE.C7*. A total of five consensus QTLs for GE were therefore detected (Table 2).

Regarding GP, a total of eight identified QTLs were detected in the three SLs, distributed across chromosomes A4, A5, C2, C3, and C4 (Table 2). They explained variances ranging from 2.31 to 16.37%, in which *iqGP/16SL.C3* and *iqGP/17SL.C3* explained 10.35% and 16.37% of the PV, respectively. Using comparative analyses, it was found that these two identified QTLs and *iqGP/15SL.C3* were co-localized in the of 28.5–30.5 cM interval of C3. These three identified QTLs were integrated into one consensus QTL (*qGP.C3*) using QTL meta-analysis. QTL *qGP.C3* was stably expressed in the three SLs and was regarded as the major QTL. The remaining five identified QTLs were also recorded as four consensus QTLs, namely *qGP.A4*, *qGP.A5*, *qGP.C2-1*, *qGP.C2-2*, and *qGP.C4*. In total, six consensus QTLs for GP were detected (Table 2).

The comparative analyses found that four of the five consensus QTLs for GE were co-located with four of the six consensus QTLs for GP (Table 2), including the major QTL *qGE.C3*. This result may indicate that GE has a marked influence on GP, and that these QTLs affect GP by regulating GE.

### 2.3. Epistatic Interactions between QTLs

Epistatic interaction mapping for GE detected six additive-by-additive epistatic effects for 12 QTLs in the three SLs (Table 3). These epistatic effects explained 3.39–13.01% of the PV, of which three explained 11.01%, 10.04%, and 13.01% of the PV, respectively. Obviously, epistatic interactions between QTLs had significant effects on seed germination in the three SLs. In terms of the value of the ‘Add-by-Add’, four are positive and two are negative, indicating that the epistatic effect comes from both parents.

Regarding GP, nine additive-by-additive epistatic effects of 18 QTLs were detected using the epistatic interaction mapping method (Table 3). The explained PV ranged from 2.41 to 10.1%. Of these nine epistatic interactions, seven were from the 16SL and two were from the 15SL. The ‘Add-by-Add’ values of all epistatic interactions between QTLs were positive, indicating that the epistatic effects come from the female parent ‘APL01’.

### 2.4. Transcriptomic Analysis of Seed Germination

A comparative analysis of the transcriptome of pre-germinated seeds (of both A0 and H0) and germinated seeds (of both A1 and H1) showed that the number of up-regulated genes during seed germination in ‘APL01’ was 5415 (A1 > A0), and the number of up-regulated genes in ‘Holly’ was 4761 (H1 > H0) (Table 4). In the pre-germination seeds of ‘Holly’ (H0), 4685 genes (H0 > A0) were up-regulated compared with the pre-germination seeds of ‘APL01’ (A0) (Table 4).

#### 2.4.1. Transcriptomic Basis of Oilseed Rape Seed Germination

**Figure 2:**
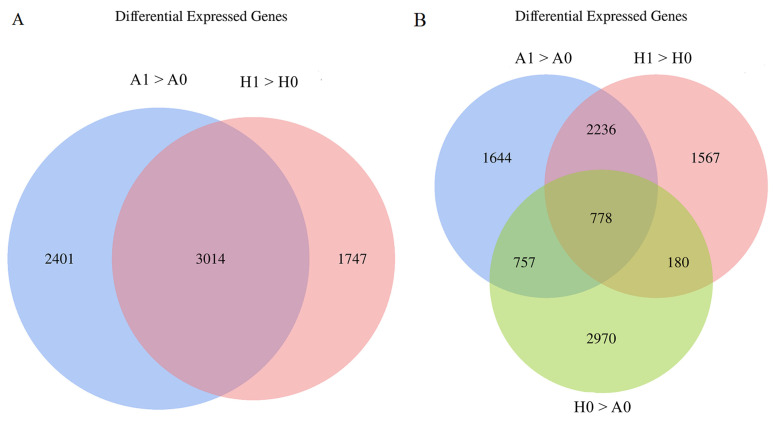
The number of differentially expressed genes in different comparison groups among the four treatment samples of ‘APL01’ and ‘Holly’. (**A**) Co-upregulated genes in the A1 > A0 and H1 > H0 groups ((A1 > A0) & (H1 > H0)). (**B**) Co-upregulated genes in the H0 > A0, A1 > A0 and H1 > H0 groups. ((H0 > A0) & (A1 > A0) & (H1 > H0)). A0 represents the pre-germination seed of ‘APL01’, while A1 represents the germinated seed of ‘APL01’. H0 represents the pre-germination seed of ‘Holly’, while H1 represents the germinated seed of ‘Holly’. A1 > A0 refers the genes up-regulated in A1 compared with A0. H1 > H0 refers to the genes up-regulated in H1 compared with H0. H0 > A0 refers to the genes up-regulated in H0 compared with A0.

Venn analysis showed that a total of 3014 differentially expressed genes (DEGs) were detected in the A1 > A0 and H1> H0 groups ((A1 > A0) & (H1 > H0)) during the seed germination process of ‘APL01’ and ‘Holly’ (Figure 2A). Gene ontology (GO) enrichment analysis revealed that these up-regulated genes were significantly enriched in 155 GO terms (Table S1). The 57 GO terms related to biological processes were mainly related to sugar, protein, lipid, amino acid, and cellular component metabolism, and signal transduction. Of the 10 GO terms related to cellular components, nine were related to cellular component metabolism, except for the ubiquitin ligase complex. Of the 88 GO terms related to molecular functions, most were related to sugar, protein, lipid and amino acid metabolism, and the tricarboxylic acid (TCA) cycle and electron transfer.

Kyoto Encyclopedia of Genes and Genomes (KEGG) enrichment analysis found that DEGs were mainly enriched in pathways related to sugar, lipid, and amino acid metabolism (Table S2), such as “amino sugar and nucleotide sugar metabolism”, “starch and sucrose metabolism”, “fatty acid metabolism”, and “cysteine and methionine metabolism”. Based on these results, the process of oilseed rape seed germination involves sugar, protein, lipid, and amino acid metabolism, and the TCA cycle, electron transport, and signal transduction, while the formation of cell structural components are coupled.

#### 2.4.2. Transcriptomic Basis of the Difference in Seed Germination between ‘APL01’ and ‘Holly’

Venn analysis detected a total of 778 DEGs in the H0 > A0, A1 > A0 and H1 > H0 groups ((H0 > A0) & (A1 > A0) & (H1 > H0)) (Figure 2B). GO enrichment analysis found that these DEGs were significantly enriched in 75 GO terms, 65 of which were consistent with the results of the analysis of the (A1 > A0) & (H1 > H0) group (Table S3). GO functional analysis found that 28 of the 75 GO terms were biological process-related terms, five were cellular process-related terms, and 42 were molecular function-related terms (Table S3). Of the biological process DEGs enriched in GO terms, most related to sugar, protein, lipid, and amino acid metabolism. The cellular component DEGs enriched in GO terms related to cell structure component metabolism and the TCA cycle. Most of the molecular function DEGs enriched in GO terms were related to sugar metabolism, the TCA cycle, and electron transfer.

KEGG enrichment analysis indicated that DEGs were mainly enriched in 16 pathways, nine of which were consistent with the results of the (A1 > A0) & (H1 > H0) group analysis, and involved sugar, lipid, cysteine, and methionine metabolism (Table S4). The remaining pathways mainly involved the biosynthesis of flavonoids, steroids, and folate. In summary, the differences between ‘APL01’ and ‘Holly’ relating to sugar, protein, lipid, and amino acid metabolism, and in the TCA cycle and electron transfer may be the molecular basis for the better germination of ‘Holly’ seeds than ‘APL01’.

Of the 1747 genes that were specifically up-regulated in H1 compared with H0 (Figure 2A), GO enrichment analysis showed that DEGs were mainly enriched in 78 GO terms, 39 of which were consistent with the results of the analysis of the (A1 > A0) & (H1 > H0) and/or (H0 > A0) & (A1 > A0) & (H1 > H0) groups (Table S5). Of these 78 GO terms, 28 were classified as biological processes mainly involving sugar, protein, lipid, amino acid and DNA metabolism, and signal transduction. The six terms related to cellular components involved DNA replication and the formation of cell structure components. The 44 terms related to molecular functions were mainly related to protein and lipid metabolism, and electron transfer and DNA replication.

KEGG enrichment analysis detected 21 pathways significantly enriched by DEGs (Table S6). These pathways were divided into two categories: the 10 pathways consistent with the results of the analysis of the (A1 > A0) & (H1 > H0) and/or (H0 > A0) & (A1 > A0) & (H1 > H0) groups, including “starch and sucrose metabolism” and “plant hormone signal transduction”; and the 11 pathways specifically detected in the H1 > H0 group, including D-Glutamine, D-glutamate, glycerophospholipid, and vitamin B6 metabolism. In summary, the genes specifically up-regulated in the H1 > H0 group were shown to not only regulate the germination of ‘Holly’ seeds through the common pathway regulating the germination of oilseed rape seeds, but also through several specific pathways regulating the germination of ‘Holly’ seeds in particular.

### 2.5. Identification of Candidate Genes

Physical mapping of the QTLs indicated that the major QTL, *qGE.C3*, was mapped to the 5,014,249–5,530,150 bp interval on the C3 chromosome of oilseed rape, and a total of 109 genes were identified from the *B. napus* genome database (http://www.genoscope.cns.fr/brassicanapus/ (accessed on 15 May 2021)) (Table S7). Transcriptome comparative analysis showed that four genes (A0 < H0) were down-regulated in A0 compared with H0, and that one gene (H1 > H0) was specifically up-regulated in H1 compared with H0 (Table 5). Comparative analyses of DNA sequences revealed that 30 of the 109 genes showed sequence variations between ‘APL01’ and ‘Holly’ (Table 5), including the gene specifically up-regulated in H1 compared to H0, *BnaC03g10380D*. The types of variations included SNPs and indels, which were distributed in the upstream and downstream regions, exons, and introns of the genes (Table S8). Combined with gene function annotation, 13 genes were identified as promising candidates for regulating the germination of *B. napus* seeds, including two involved in sugar metabolism, nine involved in plant hormone signaling, one involved in methionine biosynthesis, and one involved in protein interactions (Table 5). The sequence variation of these candidate genes between ‘APL01’ and ‘Holly’ may lead to changes in gene expression level, or loss or changes in gene function, which in turn affect seed germination.

## 3. Discussion

### 3.1. Major QTLs Couple with Minor QTLs to Co-Regulate the Germination of Brassica Napus Seeds

Seed germination is a complex trait influenced by both internal factors (sugar, protein, lipid metabolism, energy production, and signal transduction) and external factors (moisture, temperature, oxygen, and light conditions) during seed germination [1,35]. In previous studies, several genetic loci have been identified that regulate the seed germination of *B. napus* and *B. rapa*, using QTL mapping and genome-wide association analytic methods [30,31,33]. Many loci have been detected that explain more than 10% of PV, and many others which explain less than 10% of PV were also detected. For example, Basnet et al. identified four QTLs for two seed germination-related traits in *B. rapa* [30], two of which explained 14% and 15.4% of the PV, while the other two explained 7.6% and 7.3% of the PV, respectively. In this study, five consensus QTLs for GE and six consensus QTLs for GP were identified. Except for *qGE.C3* and *qGP.C3*, the other QTLs explained less than 10% of the PV, indicating that the major and minor QTLs cooperate to regulate the germination of *B. Napus* seeds, just like in *B. rapa* [30].

In addition, it is worth noting that the major QTL *qGE.C3* (*qGP.C3*) identified in this study is different from those previously reported [30,31,33], implying that it may be a new locus that regulates the germination of *B. napus* seeds. In the future, it is hoped that by developing molecular markers closely linked to this QTL, and using molecular marker-assisted selection, will improve the seed germination traits of *B. napus*.

### 3.2. Epistatic Effects between QTLs Are Involved in Seed Germination

Previous studies have shown that plant seed germination is regulated by antagonistic effects between plant hormones [2]. Epistatic interactions between QTLs have also been reported to be involved in the regulation of seed germination [30]. In this study, only one additive QTL for GE was identified in 15SL (*iqGE/15SL.C3*) and 16SL (*iqGE/16SL.C3*). However, two epistatic interactions in 15SL (accounting for 23.1% of the PV in total) and three epistatic interactions in 16SL (explaining a total of 13.3% of the PV) were detected using ICIM-EPI mapping, respectively. Furthermore, one epistatic interaction explaining 11% of the variation was also detected in 17SL. Regarding GP, two (jointly accounting for 13.7% of the total PV) and seven (jointly accounting for 20.6% of the total PV) epistatic interactions were detected in 15SL and 16SL, respectively. Taken together, these results show that epistatic effects between QTLs have a significant effect on seed germination, and may contribute to seed germination together with additive effects.

### 3.3. Material Metabolism and Energy Production Are the Biochemical Bases of Seed Germination in Brassica Napus

The catabolism of sugar in an organism can provide energy for its life activities and also provide raw materials (i.e., a carbon skeleton) for the synthesis of lipids, proteins, nucleic acids, and other biological macromolecular substances [36]. In this study, enrichment analysis of the DEGs in the (A1 > A0) & (H1 > H0) group detected several GO terms and pathways related to sugar metabolism, mainly to the biosynthesis of extracellular polysaccharides and cellulose, and to the hydrolysis of polysaccharides such as starch and sucrose. This may reflect the raw material requirements for cell wall synthesis and subsequent energy production.

Cleavage of triacylglycerol (TAG, in seed oil) by TAG lipases generates glycerol and fatty acids, and further catabolism of glycerol can provide energy during early oilseed germination [1,37,38]. Enrichment analyses showed that DEGs in the (A1 > A0) & (H1 > H0) group were also enriched in GO terms and that the pathways related to lipid metabolism in this study were mainly related to lipid and glycerol catabolism, and fatty acid biosynthesis. In fact, previous studies have found that enzymes related to lipid and glycerol hydrolysis are reactivated during early germination [38], while fatty acid β-oxidation is activated during post-germination seedling establishment [39,40,41]. All these results indicate that lipid and glycerol hydrolysis are necessary for seed germination in *B. napus*.

Proteins stored in seeds are an important source of amino acids during early germination [42]. Methionine participates in seed germination through multiple pathways [17,18,19,20]. For example, active methionine (S-adenosylmethionine) regulates the synthesis of ethylene, biotin, polyamines, and other germination regulators [24,25]. Cys and Asp are the precursors of Met and are widely involved in the regulation of seed germination [27]. In addition, Asp and glutamate are substrates for aspartate and alanine aminotransferases, which affect ATP production by participating in the respiratory pathway [43,44,45]. In this study, the DEGs involved in amino acid metabolism in the (A1 > A0) & (H1 > H0) group were mainly enriched in GO terms through methionine and glutamine biosynthesis. In addition, KEGG enrichment analysis also detected many DEGs that were enriched in tryptophan metabolism, lysine degradation, and arginine and proline metabolism. It cannot be ruled out that these amino acids are also important for seed germination.

Enzyme activity measurements [46,47,48] and proteomic analyses [49,50,51] have suggested activation of the TCA cycle during early germination. In this study, DEGs in the (A1 > A0) & (H1 > H0) group were also enriched in three GO terms related to the TCA cycle, namely the “tricarboxylic acid cycle”, “ATP citrate synthase activity”, and “isocitrate dehydrogenase (NAD^+^) activity”. Regarding those related to molecular functions, DEGs in the (A1 > A0) & (H1 > H0) group were also significantly enriched in eight GO terms involving electron transfer coupled with ATP production, including “FMN binding”, “heme binding”, “iron ion binding”, and “oxygen binding”. This implies that energy is required for seed germination. In fact, in *Arabidopsis*, a large number of enzymes involved in energy production pathways were reactivated during early germination [49,50,52].

Phospholipase D (PLD) is an important cellular phospholipid metabolic enzyme, which plays an important role in lipid signaling, adversity defense responses, and seed germination [53,54,55]. Mutations in PLDα1 and PLDδ can alleviate the inhibitory effect of ABA on seed germination [56]. Furthermore, the PLDγ gene has also been reported to regulate *Arabidopsis* seed germination under drought stress, together with ABA [57]. Protein ubiquitination participates in the regulation of seed germination by regulating the GAs signal pathway [58]. In this study, the DEGs in the (A1 > A0) & (H1 > H0) group were found to be significantly enriched in three GO terms related to plant signaling, namely the “phosphorelay signal transduction system”, “protein ubiquitination”, and the “ubiquitin ligase complex”. These results suggest that signal transduction is essential for seed germination in *B. napus*. In addition, many significant GO terms related to cellular component metabolism were also detected. These terms are important for cell proliferation and differentiation, and may be involved in hypocotyl elongation during seed germination.

### 3.4. Differences in Sugar Metabolism, Protein Metabolism, Lipid Metabolism, Amino Acid Metabolism, the TCA Cycle, Electron Transfer, Signal Transduction, and DNA Metabolism Potentially Determine the Higher Germination Rate of ‘Holly’ Seeds

In this study, the DEGs in the (H0 > A0) & (A1 > A0) & (H1 > H0) group were mainly enriched in GO terms and in pathways consistent with the results of the analysis of the (A1 > A0) & (H1 > H0) group, which ultimately affect the catabolism of polysaccharides, cellulose synthesis, proteolysis, oligopeptide transport, methionine synthesis, fatty acid synthesis, glycerol catabolism, the TCA cycle, electron transfer, and cellular component metabolism. This reflects the molecular basis of the difference in seed germination between ‘APL01’ and ‘Holly’. In addition, the KEGG enrichment analysis showed that DEGs in the (H0 > A0) & (A1 > A0) & (H1 > H0) group were also significantly enriched in pathways involving flavonoid, steroid, and folate biosynthesis. A previous study has suggested that seed flavonoids were retained, and new flavonoids were synthesized during germination in *Phaseolus vulgaris* [59]. Transcriptome and metabolome analyses revealed that germinated seeds promoted flavonoid synthesis and inhibited lignin synthesis, which could be beneficial to germination in *Polygonatum cyrtonema* seeds [60]. BR is a steroid plant growth regulator necessary for the growth and development of a new plant. BR can relieve the inhibition of seed germination caused by ABA [61]. Folate, also called vitamin B9, is involved in DNA synthesis and is therefore critical for seed germination [62]. These results indicate that the biosynthesis of flavonoids, steroids, and, potentially, folates participate in the regulation of germination in oilseed rape.

Regarding the 39 GO terms specifically enriched in the H1 > H0 group, most are related to the regulation of signal transduction and the initiation of DNA replication. Signal transduction regulation mainly involves protein phosphorylation and inositol metabolism. Previous studies have shown that several proteins involved in ABA (reviewed by Umezawa et al.) [63] and BR [64] signal transduction were phosphorylated to regulate seed germination during the early stages. In plants, inositol is involved in seed germination, sugar transport, stress responses, and cell wall biogenesis [65,66]. Therefore, differences in protein phosphorylation and inositol metabolism may also be related to the differences in germination rate between ‘APL01’ and ‘Holly’. In addition, the DEGs detected specifically in the H1 > H0 group were also enriched in “alternative oxidase activity”, indicating that there is a difference in the alternative oxidase (AOX) pathway between ‘APL01’ and ‘Holly’. In plants, respiration is usually regulated by two pathways in the mitochondria: the cytochrome oxidase pathway (COX) and the AOX pathway [67]. In cotton *Gossypium* spp., the later regulates seed germination via temperature-dependent ROS generation [12]. Thus, the significant enrichment of DEGs in “respiratory gaseous exchange by respiratory system” indicates that there may be a significant increase in oxygen absorption and carbon dioxide release in ‘Holly’ compared with ‘APL01’ during early germination, as previously reported [68,69].

### 3.5. Genes Involved in Sugar Catabolism, Plant Hormone Signal Transduction, and Methionine Biosynthesis Are Promising Candidate Genes for Seed Germination in Brassica Napus

Combining QTL mapping, RNA sequencing, genomic DNA resequencing, and gene function annotation identified 13 promising candidate genes. Gene function annotation indicates that two genes, *BnaC03g10300D* and *BnaC03g10420D*, are potentially involved in the biosynthesis of phosphoinositides (Pins). Previous studies have shown that Pins may regulate seed germination by enhancing their response to ABA stimulation [70]. Sequence variations of these two genes in ‘Holly’ may cause ‘Holly’ to be less sensitive to ABA compared with ‘APL01’. The three genes *BnaC03g10330D*, *BnaC03g10350D*, and *BnaC03g10410D* are potentially involved in protein ubiquitination. Protein ubiquitination participates in the regulation of seed germination by regulating the GAs signal pathway [58]. This may regulate seed germination through ubiquitin-protease system-mediated protein degradation. The oilseed rape gene *BnaC03g10460D* may encode a member of the Leucine-rich repeat (LRR) family of proteins. According to the function of the LRR protein in *Arabidopsis* [71], this gene may regulate seed germination through ABA-related pathways in *B. napus*. Two putative oilseed rape glycosyl hydrolase genes, *BnaC03g10320D* and *BnaC03g10370D*, may be involved in seed germination via carbohydrate hydrolysis, because 43 enzymes A and B in the glycosyl hydrolase family of *Arabidopsis* promote seed germination by releasing dormancy [72]. The oilseed rape gene *BnaC03g11340D* encodes a putative Dof protein, potentially regulating seed germination. In fact, the role of the Dof protein in seed germination has been determined in barley *Hordeum vulgare* [73]. The putative oilseed rape gene *BnaC03g10840D* encodes a member of the basic leucine zipper (bZIP) transcription factor family. In wheat *Triticum* spp., two members of the bZIP protein family, ABA-responsive element and ABA-responsive element binding factor, are important transcription factors that regulate the ABA signaling response [74]. Therefore, we speculate that this gene may participate in the regulation of seed germination via ABA signaling in *B. napus*. The putative oilseed rape gene *BnaC03g10380D* may encode an HSP20-like chaperone superfamily protein. Small-molecule heat shock proteins generate extra ROS through the AOX respiratory pathway to promote seed germination [12]. ROS may inhibit the transport of ABA from the cotyledons to the embryos, in turn promoting germination [13]. It cannot be ruled out that this gene may promote higher seed germination rate in ‘Holly’ through the AOX pathway compared with in ‘APL01’. The oilseed rape gene *BnaC03g11220D* encodes a putative lectin protein kinase family protein, which, based on the effect of protein phosphorylation on seed germination in other plants, may participate in seed germination through pathways related to ABA and BR signaling, (reviewed by Umezawa et al.) [63,64]. This putative oilseed rape *methionine synthase 3* (*MS3*) gene *BnaC03g10390D* may be involved in methionine synthesis and may positively regulate seed germination in *B. napus*, just as *MS1* functions in *Arabidopsis* [18].

Of the 13 candidate genes mentioned above, six are potentially involved in ABA signaling, three are potentially involved in GAs signaling, two are involved in glycohydrolysis, and one is involved in methionine biosynthesis. This suggests that either plant hormone signal transduction, sugar catabolism, or methionine synthesis is the key factor leading to the higher germination rate of ‘Holly’ seeds than that of ‘APL01’.

## 4. Materials and Methods

### 4.1. Plant Materials and Phenotyping

A population of *B. napus* RILs (denoted the AH-RILs population) consisting of 189 F_9_ generation lines was developed from a cross between the low-germination-rate variety ‘APL01’ and the high-germination-rate variety ‘Holly’. The seeds of this population were produced by artificially assisted self-pollination in three different environments during the growing seasons of 2014/2015, 2015/2016 and 2016/2017 in Nanjing, China (altitude: 26 m; latitude: 31.59° N; longitude: 119.19° E; silty loam soil), China. Seeds were stored at natural temperature (an average daily maximum temperature of 22 °C an average daily minimum temperature of 13 °C) and humidity (an average daily relative humidity of 76%). The genotypes of this population and two parents were analyzed using simple sequence repeat (SSR) markers and the Illumina (San Diego, CA, USA) *Brassica* 60 K Infinium array by DNA Landmarks (Saint-Jean-sur-Richelieu, QC, Canada) in a previous study [75]. A high-density single nucleotide polymorphism (SNP) linkage map incorporating 2755 bins (involving 11,458 SNPs) and 57 SSRs, covering 2027.53 cM with an average marker interval of 0.72 cM was developed for QTL mapping of the apetalous trait [75,76], seed fatty acid composition [77], flowering time [78], and stem mechanical strength [79].

The seed germination experiment was conducted in August 2018, using a completely randomized trial design with three replicates. A total of 191 lines (including 189 RILs and two parents) were used for the experiment. In each of the three replicates, 100 seeds of each line were sown on sterile filter paper in a Petri dish. Seeds were germinated in a growth chamber at a constant temperature of 20 °C with 75% relative humidity and a photoperiod of 16 h light/8 h darkness. A seed whose radicle broke through the seed coat whose length was equal to the seed diameter was defined as a germinating seed. GE and GP were assessed according to the recommendations of the International Seed Testing Association (ISTA, Bassersdorf, Switzerland, 2017). The germination rate of seeds on the 5th day after sowing was regarded as the GE, and the germination rate on the 10th day was regarded as the GP.

### 4.2. QTL Mapping

QTL mapping for GE and GP was performed using the QTL IciMapping software [80]. Mapping methods included inclusive composite interval mapping for additive QTL (ICIM-ADD) and ICIM for epistatic mapping (ICIM-EPI). For ICIM-ADD, Step and PIN values were set to 1 cM and 0.02, respectively. The logarithm of odds (LOD) thresholds for detecting a significant QTL (3.9 for GE, 3.4 for GP) were determined on the basis of permutation test analyses (1000 permutations, 5% overall error level). For ICIM-EPI, the Step, PIN, and LOD thresholds were set to 5 cM, 0.0001, and 5, respectively.

The names for the identified QTLs were formed beginning with the abbreviation ‘iq’ (i.e., identified QTL), followed by the trait abbreviation (GE or GP), seed lot (SL) of the germination experiment (15SL, 16SL and 17SL), and linkage group (A1–A10 and C1–C9). The software program BioMercator v4.2 program [81] was used for the QTL meta-analysis, and the lowest Akaike information criterion value containing model was considered the best model. The identified QTLs with overlapping CIs that were detected in different experiments were integrated into a consensus QTL. In the statistical analysis, a specific QTL detected in only one experiment was also treated as a consensus QTL. Consensus QTLs were named using the abbreviation ‘q’ (i.e., QTL), followed by the trait abbreviation (GE or GP) and linkage group (A1–A10 and C1–C9). If two or more QTLs were detected in a linkage group, they were numbered sequentially (e.g., *iqGE/17SL.C2-1*, *qGE.C2-1*). QTLs that were repeatedly detected in the three SLs and which explained more than 10% of the PV in at least two SLs were considered as major QTLs.

### 4.3. RNA Sequencing and Data Analysis

Fully mature seeds of ‘APL01’ and ‘Holly’ were used for germination experiments. The pre-germination seed samples were collected two hours before germination and soaked until all of the water had been fully absorbed. The pre-germination seed sample of ‘APL01’ was denoted as A0, and the germinated seed sample as A1. Pre-germinated seed samples of ‘Holly’ were denoted as H0, and the germinated seed samples as H1. Seeds from the three different SLs (15SL, 16SL and 17SL) were uniformly mixed and used for germination experiments, with 1000 seeds per SL. Three replicates were set up for each treatment, with 100 seeds for each replicate, and a total of 12 samples were used for RNA sequencing. Total RNA was isolated using a MagaZorb^®^ Total RNA Mini-Prep Kit (Promega, Madison, WI, USA). RNA degradation and contamination were monitored on 1% agarose gels. RNA purity was checked using a NanoPhotometer^®^ spectrophotometer (IMPLEN, Westlake Village, CA, USA). RNA concentration was measured using a Qubit RNA Assay Kit with a Qubit 2.0 Fluorometer (Life Technologies, CA, USA). Messenger RNA library construction, sequencing, and raw data processing were entrusted to Hangzhou Lianchuan Biotechnology Co., Ltd. (Zhejiang, China). An Illumina Novaseq™ 6000 was used for RNA sequencing, with sequencing read lengths of 150 bp with paired ends. The ‘Darmor-*bzh*’ genome was selected as the reference genome for *B. napus* [82]. The identification of DEGs, Venn analysis, GO and KEGG enrichment analysis were all performed using the Lianchuan Biological Cloud Platform (https://www.omicstudio.cn/tool (accessed on 25 June 2021)). For the Venn analyses, A1 > A0 refers to genes that were up-regulated in A1 compared with A0, while H1 > H0 refers to genes that were up-regulated in H1 compared with H0. H0 > A0 refers to genes that were up-regulated in H0 compared with A0. (A1 > A0) & (H1 > H0) refers to genes that were up-regulated in both the A1 > A0 and H1 > H0 groups, while (H0 > A0) & (A1 > A0) & (H1 > H0) refers to genes that were up-regulated in the H0 > A0, A1 > A0 and H1 > H0 groups.

### 4.4. Mining Candidate Genes

Based on the known location of the SNP marker harbored in the *Brassica* 60 K SNP BeadChip Array on the *B. napus* reference genome (http://www.genoscope.cns.fr/brassicanapus/ (accessed on 15 May 2021)), the QTL could be riveted to a specific position on the oilseed rape genome, and the genes within the CI of the QTL were obtained. The changes in expression of these genes during seed germination between different treatment samples of ‘APL01’ and ‘Holly’ were determined using RNA sequencing. The variations in the gene sequences between ‘APL01’ and ‘Holly’ were determined using genomic DNA resequencing in a previous study [79]. Finally, based on the predicted gene function, promising candidate genes that regulate seed germination were identified in *B. napus*.

## 5. Conclusions

Major QTLs, minor QTLs, and epistatic effects between QTLs jointly regulate the germination of *B. napus* seeds. The major QTL *qGE.C3* (*qGP.C3*) is considered to be a novel locus regulating seed germination. Either plant hormone signal transduction, sugar catabolism, or methionine synthesis is the key factor leading to the higher germination rate of ‘Holly’ seeds than that of ‘APL01’. The differences in sugar, protein, lipid, amino acid, and DNA metabolism, and the TCA cycle, electron transfer, and signal transduction between ‘APL01’ and ‘Holly’ may be a cascade effect caused by this key factor.

## Figures and Tables

**Table 1:** Summary statistics of seed germination parameters in 15SL, 16SL and 17SL.

Trait	Seed Lot	Parents		RIL Lines		Skewness	Kurtosis	Heritability
		APL01	Holly	Range	Mean ± SD			
Germination energy	15SL	72.0	92.0	0.0–100.0	77.1 ± 21.1	−1.26	0.95	80.2%
	16SL	82.0	96.0	0.0–100.0	81.3 ± 20.5	−1.71	2.80	
	17SL	90.0	98.0	4.0–100.0	81.4 ± 19.8	−1.57	2.27	
Germination percentage	15SL	72.0	92.0	0.0–100.0	81.1 ± 19.9	−1.52	1.86	80.0%
	16SL	82.0	100.0	0.0–100.0	82.5 ± 20.4	−1.76	2.93	
	17SL	90.0	100.0	6.0–100.0	83.9 ± 19.4	−1.75	2.83	

15SL refers to the seed lot harvested in 2015. 16SL refers to the seed lot harvested in 2016. 17SL refers to the seed lot harvested in 2017.

**Table 2:** QTLs that regulate seed germination detected by ICIM-ADD method.

Trait	Consensus QTL				Identified QTL							
	QTL	Chr.	Pos.	CI	QTL	Chr.	Pos.	CI	LOD	PVE (%)	Add.	Seed Lot
Germination energy	*qGE.A4*	A4	3	1.5–4.5	*iqGE/17SL.A4*	A4	3	1.5–4.5	11.42	4.50	0.09	17SL
	*qGE.C2-1*	C2	81	77.5–81.5	*iqGE/17SL.C2-1*	C2	81	77.5–81.5	9.27	3.34	0.08	17SL
	*qGE.C2-2*	C2	87	84.5–91.5	*iqGE/17SL.C2-2*	C2	87	84.5–91.5	5.32	2.20	−0.06	17SL
	*qGE.C3*	C3	29	28.5–29.5	*iqGE/15SL.C3*	C3	29	28.5–30.5	3.95	8.28	0.06	15SL
					*iqGE/16SL.C3*	C3	29	28.5–30.5	4.04	10.59	0.06	16SL
					*iqGE/17SL.C3*	C3	29	28.5–29.5	28.65	13.61	0.16	17SL
	*qGE.C7*	C7	93	92.5–93.5	*iqGE/17SL.C7*	C7	93	92.5–93.5	5.61	2.04	−0.06	17SL
Germination percentage	*qGP.A4*	A4	2	1.5–3.5	*iqGP/17SL.A4*	A4	2	1.5–3.5	3.63	2.39	0.04	17SL
	*qGP.A5*	A5	105	104.5–107.5	*iqGP/17SL.A5*	A5	105	104.5–107.5	4.38	2.48	0.04	17SL
	*qGP.C2-1*	C2	81	79.5–81.5	*iqGP/17SL.C2-1*	C2	81	79.5–81.5	19.64	2.58	0.10	17SL
	*qGP.C2-2*	C2	87	85.5–88.5	*iqGP/17SL.C2-2*	C2	87	85.5–88.5	16.06	2.31	−0.09	17SL
	*qGP.C3*	C3	29	28.5–29.5	*iqGP/15SL.C3*	C3	29	28.5–30.5	3.61	8.67	0.06	15SL
					*iqGP/16SL.C3*	C3	29	28.5–30.5	3.42	10.35	0.06	16SL
					*iqGP/17SL.C3*	C3	29	28.5–29.5	63.76	16.37	0.24	17SL
	*qGP.C4*	C4	93	92.5–93.5	*iqGP/17SL.C4*	C4	93	92.5–93.5	43.64	9.28	−0.18	17SL

Chr., chromosome. Pos., position. Add., additive. CI refers to the confidence interval of QTL.

**Table 3:** Epistatic interaction between QTLs detected by the ICIM-EPI method.

Trait	Chr._1	Pos._1	CI_1	Chr._2	Pos._2	CI_2	LOD	PVE (%)	Add._1	Add._2	Add-by-Add	Seed Lot
Germination energy	C2	85	83.5–86.5	C2	90	88.5–91.5	5.73	5.29	0.08	−0.08	0.17	16SL
	C3	100	98.5–101.5	C3	105	103.5–106.5	7.16	3.39	0.07	−0.06	0.18	16SL
	C5	25	23.5–26.5	C5	30	28.5–31.5	6.43	4.59	−0.11	0.11	0.12	16SL
	A9	65	63.5–66.5	C4	40	38.5–41.5	5.08	11.01	0.02	0.02	−0.05	17SL
	A2	40	38.5–41.5	C6	35	33.5–36.5	5.07	10.04	−0.02	0.01	−0.07	15SL
	C7	95	93.5–96.5	C8	45	43.5–46.5	6.74	13.01	−0.01	0.00	0.08	15SL
Germination percentage	A2	60	58.5–61.5	A2	65	63.5–66.5	6.88	2.98	−0.14	0.14	0.13	16SL
	C2	80	78.5–81.5	C2	85	83.5–86.5	6.19	3.30	0.14	−0.14	0.11	16SL
	C3	100	98.5–101.5	C3	105	103.5–106.5	8.43	2.41	0.06	−0.05	0.19	16SL
	C4	140	138.5–141.5	C4	145	143.5–146.5	5.34	2.47	0.14	−0.17	0.14	16SL
	C5	25	23.5–26.5	C5	30	28.5–31.5	8.28	3.35	−0.12	0.12	0.13	16SL
	C6	75	73.5–76.5	C6	80	78.5–81.5	6.62	3.29	−0.13	0.14	0.11	16SL
	C9	20	18.5–21.5	C9	25	23.5–26.5	6.32	2.83	−0.14	0.12	0.16	16SL
	C5	25	23.5–26.5	C5	30	28.5–31.5	6.00	10.10	−0.11	0.11	0.12	15SL
	C7	95	93.5–96.5	C8	45	43.5–46.5	7.05	3.60	−0.01	0.00	0.08	15SL

‘Chr._1’ refers to the chromosome where the first QTL is located. ‘Pos._1’ refers to the position of the first QTL. ‘CI_1’ refers to the confidence interval of the first QTL. ‘Add._1’ refers to the additive effect of the first QTL. ‘Chr._2’ refers to the chromosome where the second QTL is located. ‘Pos._2’ refers to the position of the second QTL. ‘CI_2’ refers to the confidence interval of the second QTL. ‘Add._2’ refers to the additive effect of the second QTL. ‘Add-by-Add’ refers to the epistatic effect between the first QTL and the second QTL.

**Table 4:** Overview of differentially expressed genes in this study.

Comparison Group	Number of Genes
A1 > A0	5415
H1 > H0	4761
H0 > A0	4685

A1 > A0 refers to the genes up-regulated in A1 compared to A0. H1 > H0 refers to the genes up-regulated in H1 compared to H0. H0 > A0 refers to the genes up-regulated in H0 compared to A0.

**Table 5:** Identification of candidate genes regulating the germination of *Brassica napus* seeds.

Gene_ID	Sequence Variation	Differential Expression	*Arabidopsis* Homologous Genes	Function Description
*BnaC03g10300D*	yes	no	*AT5G20840*	Phosphoinositide phosphatase family protein; involved in phosphoinositides biosynthesis.
*BnaC03g10310D*	yes	no	*AT5G20850*	RAS associated with diabetes protein 51 (RAD51); involved in DNA repair.
*BnaC03g10320D*	yes	no	*AT5G20870*	O-Glycosyl hydrolases family 17 protein; involved in carbohydrate hydrolysis.
*BnaC03g10330D*	yes	no	*AT5G20885*	RING/U-box superfamily protein; involved in protein ubiquitination.
*BnaC03g10340D*	yes	no	*AT5G20890*	TCP-1/cpn60 chaperonin family protein; involved in protein folding.
*BnaC03g10350D*	yes	no	*AT5G20910*	RING/U-box superfamily protein; involved in protein ubiquitination.
*BnaC03g10370D*	yes	no	*AT5G20940*	Glycosyl hydrolase family protein; involved in carbohydrate hydrolysis.
*BnaC03g10380D*	yes	H1 > H0	*AT5G20970*	HSP20-like chaperones superfamily protein; involved in ROS generation.
*BnaC03g10390D*	yes	no	*AT5G20980*	Methionine synthase 3 (MS3); involved in methionine biosynthesis.
*BnaC03g10400D*	yes	no	*AT5G20990*	B73; involved in molybdenum cofactor biosynthesis.
*BnaC03g10410D*	yes	no	*AT5G21010*	BTB-POZ and MATH domain 5 (BPM5); involved in protein ubiquitination.
*BnaC03g10420D*	yes	no	*AT5G21050*	Hyccin; involved in phosphatidylinositol phosphate biosynthetic process.
*BnaC03g10430D*	yes	no	*--*	Unknown protein
*BnaC03g10440D*	yes	no	*AT5G21080*	Uncharacterized protein
*BnaC03g10460D*	yes	no	*AT5G21090*	Leucine-rich repeat (LRR) family protein; involved in ABA signaling.
*BnaC03g10470D*	yes	no	*AT5G21130*	Late embryogenesis abundant (LEA) hydroxyproline-rich glycoprotein family
*BnaC03g10480D*	yes	no	*AT5G21160*	LA RNA-binding protein
*BnaC03g10490D*	yes	no	*AT1G32210*	DEFENDER AGAINST APOPTOTIC DEATH 1 (ATDAD1)
*BnaC03g10590D*	yes	no	*AT5G22791*	F-box family protein
*BnaC03g10620D*	yes	no	*AT5G22040*	Ubiquitin carboxyl-terminal hydrolase; involved in protein deubiquitination.
*BnaC03g10680D*	yes	no	*AT5G05760*	Syntaxin of plants 31 (SYP31); involved in cytokinesis.
*BnaC03g10800D*	no	(H0 > A0) & (A1 > A0) & (H1 > H0)	*AT5G22310*	Trichohyalin-like protein; functions in protein binding.
*BnaC03g10840D*	yes	no	*AT2G42380*	BZIP34; FUNCTIONS IN: involved in the regulation of ABA signaling response.
*BnaC03g10920D*	yes	no	*AT5G22670*	F-box/RNI-like/FBD-like domains-containing protein
*BnaC03g10950D*	no	H0 > A0	*AT5G22740*	Cellulose synthase-like A02 (CSLA02); involved in mucilage biosynthesis.
*BnaC03g10960D*	yes	no	*AT5G22750*	RAD5; involved in DNA repair.
*BnaC03g11030D*	no	(H0 > A0) & (A1 > A0) & (H1 > H0)	*AT5G22880*	Histone B2 (HTB2); involved in nucleosome assembly.
*BnaC03g11050D*	no	H0 > A0	*AT5G22920*	CHY-type/CTCHY-type/RING-type Zinc finger protein; involved in regulation of stomatal opening.
*BnaC03g11080D*	yes	no	*AT5G22950*	SNF7 family protein; involved in vesicle-mediated transport.
*BnaC03g11220D*	yes	no	*AT3G45390*	Concanavalin A-like lectin protein kinase family protein; involved in protein phosphorylation.
*BnaC03g11310D*	yes	no	*AT5G60210*	ROP interactive partner 5 (RIP5)
*BnaC03g11340D*	yes	no	*AT5G60200*	TARGET OF MONOPTEROS 6 (TMO6); involved in seed germination.
*BnaC03g11350D*	yes	no	*AT5G60190*	Cysteine proteinases superfamily protein; involved in proteolysis.

The underlined genes are promising candidate genes. For sequence variation, ‘yes’ means that there is a variation in the gene sequence between ‘APL01’ and ‘Holly’, while ‘no’ means that there is no variation in the gene sequence between ‘APL01’ and ‘Holly’. For differential expression, H1 > H0 means that the gene is up-regulated in H1 compared to H0; H0 > A0 means that the gene is up-regulated in H0 compared to A0; (H0 > A0) & (A1 > A0) & (H1 > H0) means that the gene is up-regulated in the H0 > A0, A1 > A0 and H1 > H0 groups; ‘no’ means that the gene has no differential expression in the H0 > A0, H1 > H0 and (H0 > A0) & (A1 > A0) & (H1 > H0) groups.

## Data Availability

Not applicable.

## Supplementary Materials

The following are available online at https://www.mdpi.com/article/10.3390/biom11121780/s1, Table S1: GO terms that are significantly enriched by DEGs in the (A1 > A0) & (H1 > H0) group; Table S2: Pathways that are significantly enriched by DEGs in the (A1 > A0) & (H1 > H0) group; Table S3: GO terms that are significantly enriched by DEGs in the (H0 > A0) & (A1 > A0) & (H1 > H0) group; Table S4: Pathways that are significantly enriched by DEGs in the (H0 > A0) & (A1 > A0) & (H1 > H0) group; Table S5: GO terms significantly enriched by DEGs that were specifically up-relgulated in the H1 > H0 group; Table S6: Pathways significantly enriched by DEGs that were specifically up-regulated in the H1 > H0 group; Table S7: Genes within the *qGE.C3*/*qGP.C3* confidence interval obtained from the reference genome of *Brassica napus*; Table S8: DNA sequence variation within the mapping interval of *qGE.C3*/*qGP.C3* detected by genome resequencing between ‘APL01’ and ‘Holly’.

## Abbreviations

ABA, abscisic acid; AdoMet, S-adenosylmethionine; AOX, alternative oxidase; Asp, aspartic acid; BR, brassinolide; bZIP, basic leucine zipper; CI, confidence interval; CK, cytokinin; COX, cytochrome oxidase pathway; Cys, cysteine; ETH, ethylene; GA, gibberellin; GE, germination energy; GO, gene ontology; GP, germination percentage; GSH, glutathione; IAA, auxin; ICIM-ADD, inclusive composite interval mapping for additive QTL; ICIM-EPI, ICIM for epistatic mapping; JA, jasmonic acid; KEGG, Kyoto Encyclopedia of Genes and Genomes; LRR, Leucine-rich repeat; Met, methionine; MS1, methionine synthase 1; MS3, methionine synthase; Pins, phosphoinositides; PLD, Phospholipase D; PV, phenotypic variation; QTL, quantitative trait locus; RIL, recombined inbred line; ROS, reactive oxygen species; SA, salicylic acid; SL, seed lot; SNP, single nucleotide polymorphism; SSR, simple sequence repeat; TAG, triacylglycerol; TCA, tricarboxylic acid.
